# The association between proximity to animal-feeding operations and community health: a protocol for updating a systematic review

**DOI:** 10.1186/2046-4053-3-99

**Published:** 2014-09-08

**Authors:** Annette M O’Connor, Brent W Auvermann, Julian PT Higgins, Shelley P Kirychuk, Jan M Sargeant, Susanna G Von Essen, Julie M Glanville, Hannah Wood

**Affiliations:** 1 College of Veterinary Medicine, Iowa State University, Ames, Iowa, USA; 2 Texas A&M AgriLife Research, Amarillo, Texas, USA; 3 School of Social and Community Medicine, University of Bristol, Bristol, UK; 4 Centre for Reviews and Dissemination, University of York, York, UK; 5 Department of Medicine, University of Saskatchewan, Saskatoon, Saskatchewan, Canada; 6 Centre for Public Health and Zoonoses, University of Guelph, Guelph, Ontario, Canada; 7 Department of Environmental, Agricultural and Occupational Health, College of Public Health, University of Nebraska Medical Center, Omaha, Nebraska, USA; 8 York Health Economics Consortium, University of York, York, UK

**Keywords:** Systematic review, Animal-feeding operations, Human health

## Abstract

**Background:**

Livestock and poultry operations that feed large numbers of animals are common. Facility capacity varies, but it is not uncommon for facilities to house 1,000 swine with multiple barns at a single site, feedlots to house 50,000 cattle, and poultry houses to house 250,000 hens. There is primary research that suggests livestock facilities that confine animals indoors for feeding can represent a health hazard for surrounding communities. In this protocol, we describe a review about the association between proximity to animal-feeding operations (AFOs) and the health of individuals in nearby communities. A systematic review of the topic was published by some members of our group in 2010. The purpose of this review is to update that review.

**Methods/Design:**

The populations of interest are people living in communities near livestock production facilities. Outcomes of interest are any health outcome measured in humans such as respiratory disease, gastrointestinal disease, and mental health. Measures of antibiotic resistance in people from the communities compared to measures of resistance found in animals and the environment on animal-feeding operations will also be summarized. The exposure of interest will be exposure to livestock production using a variety of metrics such as distance from facilities, endotoxin levels, and measures of odor. Electronic searches will be conducted using MEDLINE and MEDLINE In-Process (via OvidSP), CAB Abstracts (via Web of Knowledge), and Science Citation Index (via Web of Knowledge). No language or date restriction will be applied. We will access the risk of bias using a pilot version of a tool developed by the Methods Groups of the Cochrane Collaboration for non-randomized interventions.

We propose to conduct a meta-analysis for each health metric (e.g., combining all respiratory disease outcomes, combining all gastrointestinal outcomes). A planned subgroup analysis will be based on the domains of the risk of bias.

**Discussion:**

This systematic review will provide synthesis of current evidence reporting the association between living near an animal-feeding operation and human health.

**Systematic review registration:**

PROSPERO CRD42014010521

## Background

Livestock and poultry operations that feed large numbers of animals are common in the USA. Facility capacity varies greatly by region, and it is not uncommon for facilities to house 1,000 swine with multiple barns at a single site, feedlots to house 50,000 cattle, and poultry houses to house 100,000 hens. Other countries with large agricultural sectors such as Canada, Brazil, Denmark, Germany, Poland, and Australia often have similarly sized operations. Facilities with a large number of animal units (i.e., exceeding an arbitrarily established threshold of species-specific animal numbers) are frequently referred to as concentrated/confined animal-feeding operations (CAFO). Confinement facilities housing fewer animal units than the species-specific thresholds are considered animal-feeding operations (AFOs); the “AFO” classification may also be used in a more inclusive sense to refer to both CAFO and confinement facilities below the threshold(s). There is primary research that suggests livestock facilities that confine animals indoors for feeding can represent an occupational hazard for workers [1-3]. The health effects are primarily associated with respiratory system function. One systematic review of the topic was published by some members of our group in 2010 [4]. The conclusions of prior reviews often differed, likely mainly due to differences in evidence used. Some reviews included indirect forms of evidence, including correlations or extrapolation from topics with similar issues (e.g., findings from studies of communities near coal industries were considered evidence for findings of communities near AFOs). Other differences arose perhaps because some reviews excluded papers from consideration based on a potential for bias.

In the period since the publication of the systematic review, several new primary research articles have been published and a new question has arisen related to antimicrobial resistance, which has not been addressed by previous reviews. There is a widespread concern about the use of antimicrobial products for growth promotion in livestock production. Frequently, this concern is focused on the transmission of resistant organisms through the farm-to-fork pathway and its impact on public health and the reservoir of resistant organisms. The application of manure to land raises concern about the spread of organisms from animal facilities, through land and water, to communities surrounding those facilities. In this review update, we propose to include antimicrobial resistance as an eligible outcome. Prior to conducting this update, in the interest of full transparency for the scientific community, the present manuscript aims to specify and clarify the protocol by which the review will be conducted.

In the protocol, we propose to update the prior review with respect to the review question, “What is the association between animal-feeding operations and the measures of the health of individuals living near animal-feeding operations but not actively engaged in livestock production?” In the original review, the World Health Organization (WHO) definition of health was used. In this updated review, we expand that definition to include data about the antimicrobial resistance patterns of organisms cultured from individuals living near animal feeding operations.

## Methods/Design

### Study registration

This protocol has been registered with PROSPERO (registration number: CRD42014010521). The systematic review protocol will be reported using the Preferred Reporting Items for Systematic Reviews and Meta-Analyses (PRISMA) statement guidelines [5].

### Review question

“What is the association between animal-feeding operations and measures of the health of individuals living near animal-feeding operations but not actively engaged in livestock production?” The following definitions will be used:

1) Animal-feeding operations: Enterprises used to rear animals for food production on any scale.

2) Health: Health is a state of complete physical, mental, and social well-being and not merely the absence of disease or infirmity as defined by WHO. In this review we will extend this definition to include antimicrobial resistance patterns of organisms cultured from individuals living near animal-feeding operations.

3) Actively engaged: Owning or working on a livestock production facility.

### Eligibility criteria for considering studies for this review

#### Study designs eligible

Eligible studies are observational studies reporting any health outcome or measures of the resistance of resident (colonized) bacterial populations measured directly on human subjects. Experience from the prior review suggests that the majority of studied outcomes are respiratory or mental health outcomes; however, studies with other outcomes are eligible. Hypothesis-generating studies based on animal studies are not considered eligible because it is unclear how an experimental study would accurately seek to reproduce short-term and long-term effects of exposure to AFO, as no validated “model” of AFO exposure exists.

Eligible studies must include more than one unit of measurement of exposure (e.g., more than one farm per exposure group) to be included because of concerns about confounding.

Ecological studies are not eligible (i.e., studies where the unit of measurement of the outcome is a population aggregate).

#### Participants eligible

The population of interest is people living in communities near and not near animal-feeding operations that might reasonably be described as industrial. This criterion excludes studies that assess the impact of occupational exposure to livestock. An exact distance for “near” is not defined as the majority of manuscripts are not expected to include this information, and this would exclude many papers from the review. The review will not limit the definition of a livestock production facility to that used by Environmental Protection Agency definition of CAFO because, again, experience suggested that many papers would not provide sufficient information to clarify the livestock population size. The intent is to study systems similar to the landless livestock (LL) production systems as defined by the Food and Agriculture Organization (FAO). However, the FAO definition states LL production systems to be this “subset of the solely livestock production systems in which less than 10% of the dry matter fed to animals is farm-produced and in which annual average stocking rates are above ten livestock units (LU) per hectare of agricultural land” (http://www.fao.org/docrep/v8180t/v8180t0y.htm). This definition is not entirely usable because some intensive operations, in particular dairies, may be able to produce >10% of the feed fed to their animals. Further, most studies will not provide such information. For this reason, the operations are generically referred to as AFOs. Such operations may occur in many countries, but systems that appear to be grass-based, nomadic, or confined smallholder operations are not relevant to the review.

#### Exposures eligible

Studies that report any measure of exposure will be eligible. There are a large number of ways exposure can be measured, such as odor, endotoxin levels in air, and proximity (distance). All will be considered eligible, including modeled exposures based on extrapolation from empirical data.

#### Outcome measures eligible

We will extract all outcomes from eligible studies measured on humans that were compared between the exposed and unexposed categories or compared by some measure of the degree of exposure. We expect some measures of exposure may be measured in a subjective but repeatable manner, such as severity of odor. Similarly, we will not exclude studies based on the approach to measurement of the outcome and address concerns about measurement error in the risk-of-bias assessment. We will not extract outcomes, such as patterns of resistance in soil or water resources, which do not represent direct health measures in humans.

### Search methods for the identification of studies

Search strategies will be developed to capture studies relevant to the review question. A draft strategy to identify records in Ovid MEDLINE and MEDLINE In-Process is presented in Additional file 1.

The strategy comprises two concepts: a) animal-feeding operations (search lines 1–9) and b) community health (search lines 11–9) (Additional file 1). Experience from the prior review conducted in this topic area suggests that specific health outcomes, such as respiratory or gastrointestinal diseases, are best characterized during the screening process rather than built into the search strategy. Inclusion of specific health outcomes as part of the community health concept returned a large number of irrelevant records about these conditions in livestock, resulting in a volume of search results much larger than could be screened within the resource constraints of the project. It is recognized that this is a pragmatic decision that has the potential to miss eligible studies that describe only a specific outcome.

The search is not limited by language, date, or study design. Animal studies are safely removed in line 21 (Additional file 1). This search line excludes only those studies which only report on health outcomes for animals rather than for humans living in proximity. News, editorials, and letters are also excluded as these types of record are unlikely to contain original research.

Capturing concepts such as community health outcomes in a robust way is challenging due to the range of free-text and index terms that could be used to describe them. Developing a strategy to attempt to capture these concepts and adapting the strategy for other database interfaces involve inevitable trade-offs to ensure that the volume of search results returned is manageable within the context of the project. Whilst the search strategy was designed to be as sensitive as possible within the time and resource constraints, it is unlikely that any strategy would be able to fully capture these concepts and there is always the risk of potentially missing relevant studies. However, the additional search techniques (such as checking reference lists and citation searching) that will be used will somewhat mitigate the risk of missing relevant studies by providing an alternative method to retrieve any eligible records that were missed by the bibliographic database searches.

The recall of the strategy has been tested against the studies included in the prior version of the review and is able to retrieve all of the studies that are indexed in MEDLINE. This suggests that it is sufficiently sensitive.

Electronic searches will be conducted using MEDLINE and MEDLINE In-Process (via OvidSP), CAB Abstracts (via Web of Knowledge), and Science Citation Index (via Web of Knowledge). The search strategy will be adapted appropriately for each search resource, taking into account differences in search syntax and indexing.

In addition to searching bibliographical databases, the reference lists of included studies will be hand-checked to identify any additional studies that may otherwise have been missed. Citation searches on eligible studies will also be undertaken using Science Citation Index and Google Scholar, as appropriate. Finally, should the searches identify any evidence published in non-peer-reviewed sources (such as theses or conference proceedings), searches will be conducted in MEDLINE, CAB Abstracts, and Science Citation Index for the first author to retrieve any associated journal or book publications.

Search results will be loaded into EndNote bibliographic management software and de-duplicated using appropriate algorithms.

All search strategies and search results will be fully documented, and this will be provided in the final report to meet the systematic review requirements for clear formal reporting of the search process.

### Selection of eligible studies

We will upload search results into online systematic review software (DistillerSR®, Ottawa, ON, Canada). In the first round of screening, abstracts and titles will be screened for inclusion. The reviewers will be DVMs with post-graduate training in epidemiology and experience with systematic review methodology. Two reviewers will independently evaluate each citation for relevance using the following questions:

1. “Does the title and/or abstract describe primary research reporting the association between livestock and human interactions (direct or indirect) and measures of human health measured on humans?”

2. “Does the study use a unit of analysis at the individual human level?”

3. “Does the study include more than one unit of measurement of exposure?”

At the first level relevance screening, reviewers will be unaware of the journal or author.

Citations will be excluded if both reviewers responded “no” to either of the questions. Although titles and abstracts not written in English will not be considered, non-English papers with English titles and abstracts will be included in the relevance screening. Relevant papers not written in English will be translated based on the number of such papers and the adequacy of funding to underwrite the translation services. The prior review identified one relevant publication published in German, and a translation of this paper is already available. Following title/abstract screening, eligibility will be assessed through full-text screening. Prior to both abstract and full-text screenings, the reviewers responsible for data extraction will undergo training to ensure consistent data extraction. At all levels of screening, any disagreements will be resolved by consensus between the two reviewers. If consensus cannot be achieved between the two reviewers, a third reviewer (AMOC or JMS) will arbitrate.

### Data collection from eligible studies

For each study, we will extract the study year, the time frame the study was conducted, the location of the study population (country), and the study location area (region within country).

For the participants, we will extract the size of the human population in the source population (when reported), the size of the study population (the number of people with metrics), and the relevant demographic information (aggregated measures of age, sex, and socioeconomic status).

For the exposure assessment, we will extract the size of the animal population in the source population. This is likely only available in general terms (e.g., type of animal, number of animals in a facility, or density in a county). We will also extract the metric(s) and units used to measure an exposed or unexposed person in the study (e.g., distance from facility, odor, and endotoxin levels).

For the outcome measures reported, we will extract either the group-level metric (exposed and unexposed people) or an effect size estimate comparing the groups (such as a regression coefficient or a function of a regression coefficient, such as an odds ratio). For all outcomes, we will extract a measure of precision and the number of people included in each category. Note that it is common that some outcome metrics are measured on all participants, and then a subset of people are subject to a more sensitive or different test. Therefore, the sample size for each outcome can vary.

We will also extract information about the approach to analysis. Experience suggests that regression models are common, and models may be adjusted or unadjusted for known confounders. Therefore, we propose to extract information about confounders studied or assessed and confounders included in final adjusted models. We also propose to purposefully extract information about any effect modifiers assessed and the findings of the assessment. If an effect modifier is significant and the data are presented for each level of that effect modifier, separate data extraction will occur for each level of the effect modifier.

### Risk-of-bias assessment for eligible studies

We will use a pilot version of a tool in development by the Methods Groups of the Cochrane Collaboration that addresses the major sources of bias for observational studies that are likely applicable to questions of bias in studies of etiology [6]. The risk-of-bias assessment will be conducted by two reviewers with training in epidemiology. We will explicitly assess the following bias domains:

• Bias due to confounding

• Bias in selection of participants into the study

• Bias in measurement of interventions

• Bias due to departures from intended interventions

• Bias due to missing data

• Bias in measurement of outcomes

• Bias in selection of the reported result

Each bias domain will be assessed as follows:

(1) Low risk of bias (the study is comparable to a well-performed randomized trial with regard to this domain);

(2) Moderate risk of bias (the study is sound for a non-randomized study with regard to this domain but cannot be considered comparable to a well-performed randomized trial);

(3) Serious risk of bias (the study has some important problems in this domain);

(4) Critical risk of bias (the study is too problematic in this domain to provide any useful evidence); and

(5) No information on which to base a judgment about risk of bias for this domain.

Where relevant, the risk of bias will be assessed for each outcome.

With respect to selection bias, it is critical that the studies be assessed for the potential to enroll participants based on exposure and outcome. Low-risk studies would be identified as those that include an approach to enrolling participants that are unrelated to a particular subgroup of the study populations of interest. Four populations of interest should have equal potential to enroll in the study; therefore, studies that purposefully recruit individuals from one group will not be eligible for inclusion. The four populations are as follows:

• Community-based individuals exposed to livestock production facilities emissions and by-products (through land/water/air) with adverse health outcomes,

• Community-based individuals exposed to livestock production facilities emissions and by-products (through land/water/air) without adverse health outcomes,

• Community-based individuals not exposed to livestock production facilities emissions and by-products (through land/water/air) with adverse health outcomes,

• Community-based individuals not exposed to livestock production facilities emissions and by-products (through land/water/air) without adverse health outcomes.

An example of a critical risk of bias for selection of participants would be as follows: “The sample consisted of two groups. The first group was a snowball sample of respondents who lived near industrial hog farms and had been identified by local grassroots activists as individuals who were distressed about the effects of the nearby hog farms” [7].

An example of a critical risk of bias for confounding would be the use of only one unit of concern for each exposure category. However, this bias has been used as an exclusion criterion (see screening questions).

### Confounding domains relevant to all or most studies

Important confounding domains include 1) family history of allergies or respiratory disease, 2) presence of wood-burning stoves, 3) exposure to tobacco smoke, 4) socioeconomic status (which may be indicated by race for some studies), 5) concurrent disease, and 6) presence of cats in the house.

### Possible co-interventions/exposures that could differ between intervention groups and could have an impact on study outcomes

Co-exposures that could differ between exposure groups are different school facilities and different industries such as grain farming or grain milling. Some industries invariably are also associated with agriculture. In particular, selection of non-rural control comparisons would mean that these important co-exposures that could be associated with the outcome would be unevenly distributed across the groups. The impact of this would be to bias away from the null. Also school facilities, particularly in rural areas, may be poorly developed and old, compared to those in urban areas; therefore, factors in school environments associated with the development of respiratory disease in children may be unevenly distributed (e.g., due to disparities in the age, design, and/or quality of ventilation systems). Also, access to health care in rural areas may be limited; therefore, severity of mental disease may differ in groups based on rural versus urban rather than exposure to AFOs.

### Process for data extraction

Prior to data extraction from eligible studies, a data extraction form will be created and pilot-tested by the data extractors and a panel member (AMOC) on a subset of studies. The extraction form will be modified as necessary based on feedback from the extractors to improve usability and ensure completeness. Data extraction will be completed independently and in duplicate. If disagreements arise, they will be resolved by consensus. If a consensus cannot be reached between the two parties, a third independent reviewer will arbitrate (AMOC, JMS). If the question relates to accuracy of the measurement of an outcome or exposure, the relevant expert will be consulted for guidance. If the question relates to a statistical approach to analysis, the relevant expert will be consulted.

After data extraction, eligible studies and extracted data will be randomly assigned to members of the panel to verify the accuracy of the extracted data. Based on the prior review and scoping, we expect each panel member will verify four to six papers. Manuscripts will be allocated to panel members using a blocked random number generator, ensuring each reviewer receives the same number of manuscripts and each manuscript has two external validators. The only exception to this approach would be if a manuscript was published in a non-English language in which one of panel members is fluent. In this situation, we would purposefully allocate the original manuscript to that reviewer and randomly allocate the translated copy to another reviewer. Prior to sending the papers to the panel members, we will mask the title, author, and journal information by redacting the PDF file accordingly. However, some manuscripts will be well known to some reviewers and likely recognizable.

If the data presented in the studies are unclear, missing, or presented in a form that is either not extractable or difficult to reliably extract, the authors of the study will not be contacted for clarification unless the paper is less than 5 years old. The cutoff for contacting authors is based on the increased likelihood of making successful contact with authors of more recently published papers. Inability to extract data will be noted in the final report, and the reason for the inability will be provided. When multiple versions of the same study are available, we will use all sources to obtain the most complete data extraction.

### Strategy for data synthesis

To combine the results from multiple studies, we will prepare an evidence profile table and summary of findings table using the GRADE approach [8-11]. However, this will be modified to address the fact that the review question relates to etiology rather than assessment of an intervention.

To summarize the findings, we propose to conduct a meta-analysis using the R package or REVMAN. A meta-analysis is planned for each health metric (e.g., combining all respiratory disease outcomes, combining all gastrointestinal outcomes). A planned subgroup analysis will be based on the domains of the risk of bias. Each study outcome will have been classified by each risk-of-bias item, and subgroup analyses for each health metric are planned. We will assess and report the association between the metric and the subgroup category. If data are sufficient, we will also explore heterogeneity using meta-regression methods. We propose also to conduct an assessment of small study effects within health outcomes and across all outcomes.

The GRADE evidence profile tables will be developed by the panel members at a 1.5 day meeting. At the meeting, the panel members will make assessments of the evidence profile. Panel members will be expected to have read the papers included in the review, the data extraction provided by the review team, and the summary of findings table.

### Strategy for presentation of the results

We will provide tables that describe the human source population, the animal source population, the outcomes measured, and the metrics of exposure.

For eligible studies, we propose to categorize the health outcome by organ system. For each of these subgroups, we will further organize the data by health outcome and exposure metric. We propose a figure/tables that will summarize the associations, stratified by the risk-of-bias results for information bias.

In the results tables, we will report the group-level characteristics (e.g., percent of people with asthma in the exposed and percent of people with asthma in the unexposed) or the effect size reported, which will likely be the odds ratio or risk ratio. We will provide information about confounders adjusted for in the estimate or if the estimate is unadjusted.

We will also provide the risk-of-bias assessment for each study by outcome included. As there may be multiple outcomes per study, risk of bias needs to be assessed at the outcome level.

## Discussion

This systematic review will provide synthesis of current evidence reporting the association between living near an animal feeding operation and human health. Results will be helpful for making public health officials and planning officials who need a transparent summary of the current state of evidence in this area.

## Competing interests

Dr. O’Connor serves as a panel member for the National Pork Board pre-harvest food safety grant program. Dr. O’Connor has received funding for primary research in pre-harvest food safety and to conduct research synthesis projects, including the prior review from the National Pork Board. The other authors (Auvermann, Glanville, Higgins, Kirychuk, Sargeant, Von Essen, and Wood) have no competing interests to declare.

## Authors’ contributions

AMOC will serve as review leader and panel organizer and, therefore, responsible for the coordination of the review, including first- and second-level relevance screening. AMOC will participate in quality assessment, data extraction, and preparing all drafts of the review protocol and the final review. JMG and HW will design and conduct the literature search and provide the report on the search results. BWA, SGVE, SPK, and JMS will all serve as panel members and participate in validating the quality assessment and data abstraction conducted by the review team. JH will act as a consultant for the conduct of the review and a resource for addressing questions related to statistical analyses of the relevant studies and assessment of risk of bias. All authors except AMOC will participate in the voting for the evidence profile and will comment on drafts of the review protocol and the final review. All authors read and approved this final manuscript.

## Supplementary Material

Additional file 1 **Draft strategy designed to identify eligible studies in Ovid MEDLINE and MEDLINE In-Process.** This file contains details of the draft search strategy used to develop the search. Click here for file
