# Supplementary material for: The association between proximity to animal-feeding operations and community health: a protocol for updating a systematic review
Source: Syst Rev. 2014 Sep 8;3:99. doi: 10.1186/2046-4053-3-99 (PMC4160559; doi:10.1186/2046-4053-3-99)
Supplement: Additional file 1 — Draft strategy designed to identify eligible studies in Ovid MEDLINE and MEDLINE In-Process. This file contains details of the draft search strategy used to develop the search. [file 2046-4053-3-99-S1.docx]

1 Animal Husbandry/ (14954)

2 Housing, Animal/ or Animal Feed/ (43800)

3 ((animal$1 or bovine or cow or cows or cattle or beef or pig or pigs or piglet$ or pork or swine or porcine or hog or hogs or finisher$ or sheep or murine or lamb or lambs or poultry or chicken$ or hen or hens or broiler$ or turkey$ or livestock or live stock or intensiv$ or industrial$ or confined or confinement or concentrated or large-scale) adj4 (feed$ operation$ or feed$ facilit$)).ti,ab. (274)

4 (cafo or cafos or afo or afos).ti,ab. (497)

5 (feed lot$1 or feedlot$ or feedyard$ or feed yard$).ti,ab. (2281)

6 ((animal$1 or bovine or cow or cows or cattle or beef or pig or pigs or piglet$ or pork or swine or porcine or hog or hogs or finisher$ or sheep or murine or lamb or lambs or poultry or chicken$ or hen or hens or broiler$ or turkey$ or livestock or live stock) adj (operation$ or facility or facilities or confined or confinement)).ti,ab. (1512)

7 ((confined or confinement) adj3 (feed or feeding)).ti,ab. (106)

8 ((intensive or intensively or large-scale or industrial) adj3 (farm or farms or farming or livestock or live stock)).ti,ab. (874)

9 ((animal production or livestock production or live stock production) adj (operation$ or facility or facilities)).ti,ab. (59)

10 or/1-9 (59163)

11 Environmental Health/ (12237)

12 environmental exposure/ or inhalation exposure/ (62750)

13 environmental pollutants/ or exp air pollutants/ or water pollutants/ (98415)

14 Environmental Illness/ (926)

15 Environmental Monitoring/ (67642)

16 (public health$ or environmental health$ or environmental medicine or community health$).ti,ab,jn,jw. (294344)

17 ((community or communities or resident$ or residence$1 or neighbor$ or neighbour$ or family or families or local$1 or populace$1 or school$1 or preschool$ or highschool$ or nursery or nurseries or playgroup$ or play group$ or kindergarten$) adj5 (health or disease$1 or impact$ or effect$1 or exposure$1 or expose$1 or outcome$1 or symptom$1 or risk$1)).ti,ab. (209189)

18 ((public or community or communities or resident$ or residence$1 or living or neighbor$ or neighbour$ or family or families or local$1 or population$1 or populace or school$1 or preschool$ or highschool$ or nursery or nurseries or playgroup$ or play group$ or kindergarten$) adj5 (proximity or vicinity or location$1 or located or nearby or near or close or closely)).ti,ab. (30362)

19 or/11-18 (664273)

20 10 and 19 (3035)

21 exp animals/ not humans/ (3934711)

22 (news or editorial or letter).pt. (1354550)

23 foot ortho$.ti,ab. (1135)

24 20 not (21 or 22 or 23) (1306)

25 remove duplicates from 24 (1295)
